# ACE2 correlated with immune infiltration serves as a prognostic biomarker in endometrial carcinoma and renal papillary cell carcinoma: implication for COVID-19

**DOI:** 10.18632/aging.103100

**Published:** 2020-04-27

**Authors:** Jing Yang, Hongxia Li, Shengda Hu, Yafeng Zhou

**Affiliations:** 1 Department of Cardiology, The First Affiliated Hospital of Soochow University, Suzhou 215006, China

**Keywords:** COVID-19, SARS-CoV-2, ACE2, immune infiltration

## Abstract

Angiotensin-converting enzyme 2 (ACE2) is a member of the renin-angiotension system, however, the correlation between ACE2 and prognosis in UCEC (Uterine Corpus Endometrial Carcinoma) and KIRP (Kidney Renal Papillary Cell Carcinoma) is not clear. We analyzed the expression levels of ACE2 in the Oncomine and TIMER databases, the correlation between ACE2 and overall survival in the PrognoScan, GEPIA and Kaplan-Meier plotter databases. The correlation between ACE2 and immune infiltration level and the type markers of immune cells was investigated in TIMER database. A prognosis analysis based on the expression levels of ACE2 was further performed in related immune cells subgroup. The ACE2 promoter methylation profile was tested in the UALCAN database. In addition, we used GSE30589 and GSE52920 databases to elucidate the changes of ACE2 expression in vivo and in vitro after SARS-CoV infection. ACE2 was elevated in UCEC and KIRP, and high ACE2 had a favorable prognosis. The expression of ACE2 was positively correlated with the level of immune infiltration of macrophage in KIRP, B cell, CD4+T cell, neutrophil and dendritic cell immune infiltration levels in UCEC. ACE2 was significantly positively correlated with the type markers of B cells and neutrophils, macrophages in UCEC, while ACE2 in KIRP was positively correlated with the type markers of macrophages. High ACE2 expression level had a favorable prognosis in different enriched immune cells subgroups in UCEC and KIRP. And the promoter methylation levels of ACE2 in UCEC and KIRP were significantly reduced. What’s more, we found that the expression of ACE2 decreased in vivo and in vitro after SARS-CoV infection. In conclusion, ACE2 expression increased significantly in UCEC and KIRP, elevated ACE2 was positively correlated with immune infiltration and prognosis. Moreover, tumor tissues may be more susceptible to SARS-CoV-2 infection in COVID-19 patients with UCEC and KIRP, which may worsen the prognosis.

## INTRODUCTION

UCEC (Uterine Corpus Endometrial Carcinoma) is a common gynecological cancer in the world [1–3]. It is an epithelial malignant tumor of endometrium, which has a high mortality rate and seriously threatens the health of women [4, 5]. It can be divided into two types: estrogen dependent and non estrogen dependent [6]. The incidence of non estrogen dependent tumors is low, but the malignancy is high and the prognosis is poor [4, 7]. The prognoses of endometrial cancer patients with metastasis are poor regardless of grade or stage, and the overall survival rate of patients is significantly reduced [8]. There is evidence that microsatellite unstable endometrial cancer has infiltration of granzyme B + cells, activated cytoxic T-lymphocytes, and PD-L1 + cells [9], which suggests that endometrial cancer can be treated with immunotherapy to improve prognosis.

KIRP (Kidney Renal Papillary Cell Carcinoma) accounts for 15%-20% of renal cancer [10]. KIRP is a malignant parenchymal tumor of the kidney, which is characterized by a papillary or tubular papillary structure [11]. It can be divided into type 1 and type 2 according to the histologic features, and type 2 KIRP has a high grade, a late stage and a poor prognosis [12, 13]. Moreover, the immune cell response is closely connected with the clinical prognosis of KIRP, and tumor related macrophages can represent the indicator of good prognosis of KIRP [14, 15]. Thus, it is necessary to clarify the relationship between UCEC and KIRP and immune invasion, and find an immune related biomarker to indicate the prognosis of UCEC and KIRP.

Angiotensin-converting enzyme 2 (ACE2) is a member of the renin-angiotension system. It’s open reading frame encodes a polypeptide containing 805 amino acids [16]. The extracellular surface of ACE2 enzyme contains a catalytic metal peptidase domain, which has 42% sequence homology with the N-terminal catalytic domain of ACE [17]. ACE2 mainly splits angiotensin II (ANG II) into angiotensin-(1-7) and acts as a vasodilator in the renin-angiotension system [18]. A recent study has shown that it can block the angiogenesis, tumor cell growth and metastasis of pancreatic cancer, breast cancer and colon cancer [19–21]. But the related prognosis and possible immune mechanisms of ACE2 in UCEC and KIRP are still ambiguous.

Since December 2019, coronavirus disease 2019 (COVID-19) was found in Wuhan City, Hubei Province, China [22]. It is caused by severe acute respiratory syndrome coronavirus 2 (SARS-CoV-2, previously tentatively named 2019-nCoV), which belongs to the beta coronaviruses (β-CoV) genus [23]. SARS-CoV-2 and severe acute respiratory syndrome coronavirus (SARS-CoV) have 79.5% homologous sequences, and it uses ACE2 as receptor to enter the cell like SARS-CoV [24]. As of March 4, 2020, there were 80409 laboratory confirmed cases and 3012 dead cases of COVID-19 in China [25]. It has been widely spread all over the world, and has been recognized as a public health emergency of international concern by the World Health Organization [26]. However, the prognosis of COVID-19 patients with UCEC and KIRP are still unclear.

In this study, we first analyzed the expression of ACE2 in different tumors in the oncomine and Tumor Immune Estimation Resource (TIMER) databases, and then used the PrognoScan, GEPIA and Kaplan-Meier plotter databases to study the prognostic relationship between ACE2 and various tumors. After screening tumors prognosis related to ACE2, the relationship between ACE2 and immune infiltration levels in different tumors was investigated in the Timer database. The ACE2 promoter methylation profile was also tested in the UALCAN database. Besides, we made use of the GSE30589 and GSE52920 databases to clarify the changes of ACE2 in cells and animals following SARS-CoV infection. Our findings shed light on the important role of ACE2 in UCEC and KIRP and also provided a potential mechanism related to immune infiltration in these tumors. It also illustrated the possible susceptibility of tumors to SARS-CoV-2 and prognosis of COVID-19 patients with UCEC and KIRP.

## RESULTS

### The mRNA expression levels in human cancers

**Figure 1:**
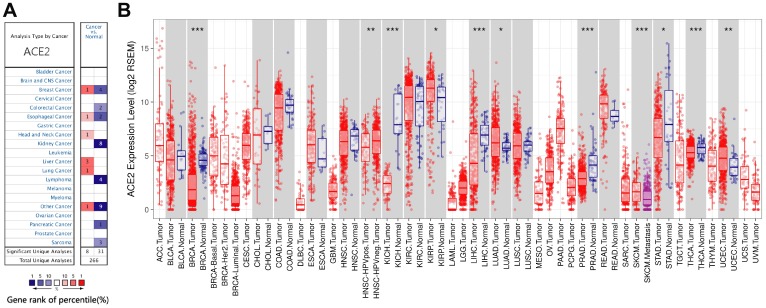
**The expression levels of ACE2 in different cancers.** (**A**) ACE2 in different cancers compared to normal tissues in the Oncomine database. (**B**) ACE2 expression levels of different tumor types in the TCGA database were detected by TIMER (^*^P<0.05, ^**^P<0.01, ^***^P<0.001).

In order to study the changes of ACE2 expression levels in different tumor tissues compared with normal tissues. We first analyzed Oncomine database, 11 databases including 739 samples were selected. The analysis showed that the expression levels of ACE2 in Invasive Breast Carcinoma, Esophageal Cancer, Head and Neck cancer, Liver cancer, Lung cancer and other cancer (Testicular Intratubular Germ Cell Neoplasi) increased significantly, while in breast cancer (Intraductal Cribriform Breast Adenocarcinoma and Invasive Breast Carcinoma), colorectal cancer, Esophageal Cancer, kidney cancer, Lymphoma, other cancer (Yolk Sac Tumor, Seminoma, Mixed Germ Cell Tumor, Embryonal Carcinoma, Testicular Embryonal Carcinoma, Testicular Yolk Sac Tumor, Testicular Seminoma, Uterine Corpus Leiomyoma, Malignant Fibrous Histiocytoma), pancreatic cancer and sarcoma decreased significantly (Figure 1A). The detailed results were summarized in Supplementary Table 1.

Then, we further studied the expression levels of ACE2 between different tumors and normal tissues based on the RNA-seq data of malignant tumors in TCGA database. The expression levels were higher in KIRP (Kidney Renal Papillary Cell Carcinoma) and UCEC (Uterine Corpus Endometrial Carcinoma) (Figure 1B). In addition, the expression level was also higher in LUAD (Lung Adenocarcinoma) (Figure 1B). Nevertheless, the expression levels of ACE2 were lower in BRCA (Breast Invasive Carcinoma), KICH (Kidney Chromophobe), LIHC (Liver Hepatocellular Carcinoma), PRAD (Prostate Adenocarcinoma), STAD (Stomach Adenocarcinoma) and THCA (Thyroid Carcinoma) (Figure 1B). We analyzed the above databases and found that the expression levels of ACE2 in breast cancer, eophagal cancer, kidney cancer, liver cancer and sarcoma were different due to different subtypes, most of which were lower than that in normal tissues except for liver cancer. What^,^ s more, ACE2 acts as a receptor for SARS-CoV-2 to enter cells, which means that tumor tissues that highly express ACE2 may be susceptible to SARS-CoV-2 infection.

### ACE2 predicted prognosis in different cancers

According to the difference of ACE2 expressions in some tumors, we further analyzed the relationship between ACE2 expression and prognosis in these tumors, so it is necessary to clarify whether ACE2 is the promoter or suppressor of tumors. PrognoScan was first used to study the relationship between the expression of ACE2 and the overall survival rate of different tumors. The analysis results showed that the high expression of ACE2 in breast cancer was related to the poor prognosis. However, the high expression of ACE2 in renal cell carcinoma had a favorable prognosis (Supplementary Table 2).

Then we analyzed TCGA database by GEPIA and explored the potential prognostic relationship between ACE2 expressions and human tumors. Interestingly, there was no significant relationship between the expressions of ACE2 and the prognosis of breast invasive carcinoma, kidney chromophobe, prostate adenocarcinoma, stomach adenocarcinoma, thyroid carcinoma, colon adenocarcinoma and head and neck squamous cell carcinoma (Supplementary Figure 1A–1G).

**Figure 2:**
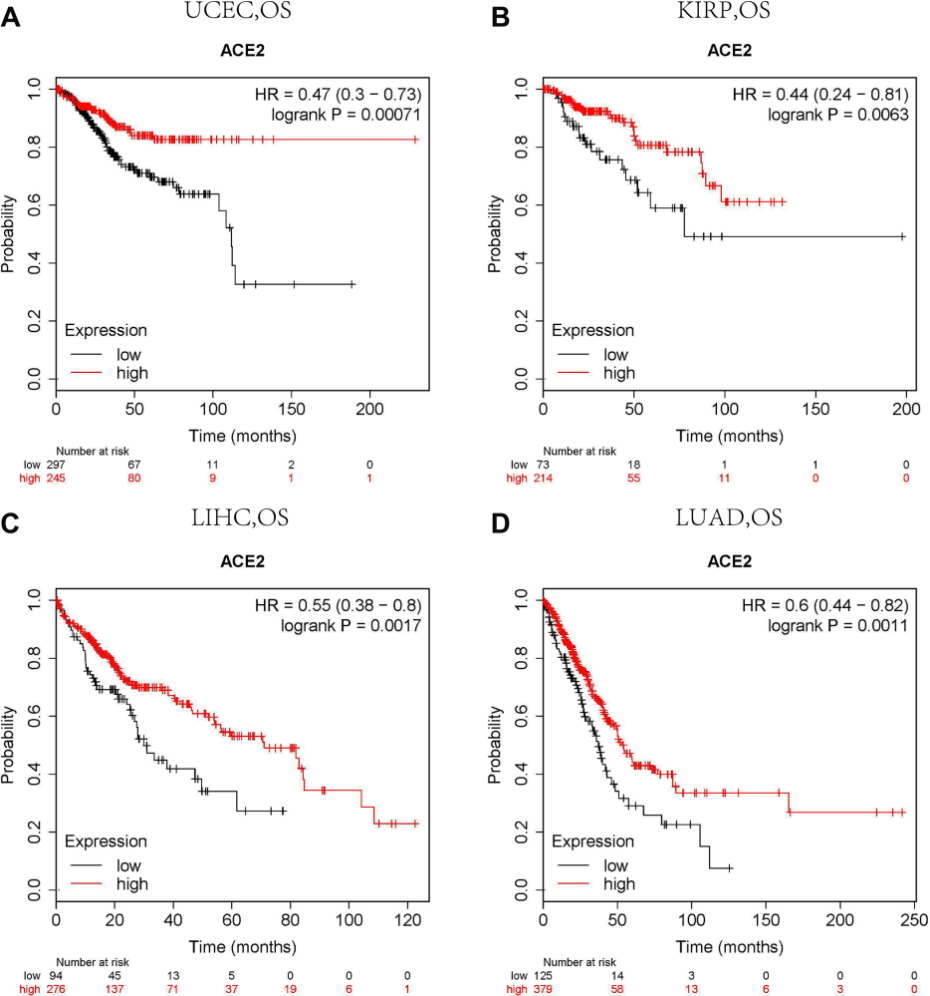
**Comparison of Kaplan-Meier survival curves of ACE2 overexpression and underexpression in different cancers.** (**A**) High ACE2 expression in the Kaplan Meier plotter database had favorable OS in UCEC (n=543), (**B**) KIRP (n=288), (**C**) LIHC (n=371) and (**D**) LUAD (n=513). OS, overall survival; UCEC, Uterine Corpus Endometrial Carcinoma; KIRP, Kidney Renal Papillary Cell Carcinoma; LIHC, Liver Hepatocellular Carcinoma; LUAD. Lung Adenocarcinoma.

The Kaplan Meier plotter is a large database containing GEO, EGA and TCGA. It can be used as a tool to evaluate genes on survival in 21 cancer types. Therefore, we used the Kaplan Meier plotter to further check the relationships between ACE2 and prognoses of different tumors. ACE2 expressions have no significant correlations with the prognoses of breast cancer, head neck squamous cell carcinoma, stomach adenocarcinoma and thyroid carcinoma (Supplementary Figure 1H–1K). However, high ACE2 expression levels in uterine corpus endometrial carcinoma and kidney renal papillary cell carcinoma showed significant favorable prognoses (OS HR0.47, 95%CI=0.30 to 0.73, OS HR0.44, 95%CI=0.24 to 0.81, respectively) (Figure 2A, 2B). Similar prognoses were also observed in liver hepatocellular carcinoma and lung adenocarcinoma (Figure 2C, 2D).

### The transcription levels of ACE2 were correlated with tumor immune infiltration

Previous studies had shown that tumor infiltration was related to the prognoses of renal cancer and endometrial cancer [9, 14, 15]. So, we tested whether the transcription levels of ACE2 in different tumors were correlated with immune infiltration. TIMER database was used to analyze the correlations between ACE2 level and uterine corpus endometrial carcinoma, kidney real papillary cell carcinoma, Liver hepatocellular carcinoma and lung adenocarcinoma. The results showed that ACE2 was very weakly negatively correlated with B cell, CD4 + T cell, dendritic cell and neutrophil of Liver hepatocellular carcinoma and lung adenocarinoma (Supplementary Figure 2).

**Figure 3:**
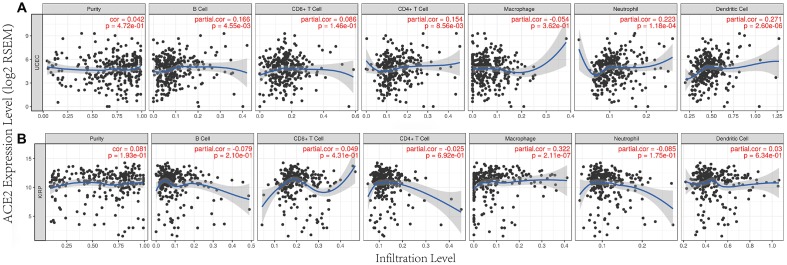
**Correlation between ACE2 expression and immune infiltration in UCEC and KIRP in TIMER database.** ACE2 expressions were positively correlated with (**A**) B cel, CD4 + T cell, neutrophil and dendritic cell immune infiltration levels of uterine corpus endofamilial carcinoma (UCEC), (**B**) the level of immune infiltration of macrophage in kidney renal papillary cell carcinoma (KIRP).

However, the expression of ACE2 was positively correlated with the level of immune infiltration of macrophage (r=0.322, p<0.001) in kidney renal papillary cell carcinoma. Similarly, ACE2 has a positive correlation with B cell (r=0.166, p<0.01), CD4 + T cell (r=0.154, p<0.01), neutrophil (r=0.223, p<0.001) and dendritic cell (r=0.271, p<0.001) immune infiltration levels of uterine corpus endometrial carcinoma (Figure 3).

### ACE2 expressions were correlated with immune cell type markers

We further explored the relationships between the expressions of ACE2 and the type markers of different immune cells in endometrial and renal carcinoma. The type markers of B-cells, CD8 + T cells, neutrophils, macrophages, dendritic cells NK cells, Th1 cells, Treg cells and monocyte were analyzed by TIMER database.

The results showed that ACE2 in UCEC was positively correlated with FCRL2 and MS4A1 in B cells. ACE2 in UCEC was also positively correlated with FCGR3B, CEACAM3, SIGLEC5, CSF3R, S100A12 in neutrophils and CD84 in macrophages (Table 1). Similarly, ACE2 in KIRP was positively correlated with CD68 and CD84 in macrophages and C3AR1 in monocyte (Table 1). These correlations remained unchanged after tumor purity and age correction (Table 1). This further confirmed that ACE2 expressions in uterine corpus endofamilial carcinoma and kidney renal papillary cell carcinoma were correlated to immune infiltration.

**Table 1:** Correlation analysis between ACE2 and immune cell type markers in TIMER database.

**Cell type**	**Gene markers**	**UCEC**						**KIRP**					
		**None**		**Purity**		**Age**		**None**		**Purity**		**Age**	
		**COR**	**P**	**COR**	**P**	**COR**	**P**	**COR**	**P**	**COR**	**P**	**COR**	**P**
B cells	FCRL2	0.325	6.7E-15	0.290	4.4E-07	0.331	2.9E-15	-0.046	4.4E-01	-0.072	2.5E-01	-0.044	4.6E-01
	CD19	0.151	4.2E-04	0.153	8.9E-03	0.159	2.1E-04	-0.040	5.0E-01	-0.026	6.8E-01	-0.040	5.1E-01
	MS4A1	0.210	7.9E-07	0.231	6.6E-05	0.217	3.6E-07	0.043	4.7E-01	0.059	3.4E-01	0.050	4.1E-01
CD8+ T cells	CD8A	0.150	4.6E-04	0.152	9.4E-03	0.149	5.0E-04	0.059	3.2E-01	0.038	5.5E-01	0.064	2.9E-01
	CD8B	0.161	1.7E-04	0.159	6.5E-03	0.158	2.3E-04	0.021	7.2E-01	0.007	9.1E-01	0.023	7.0E-01
Neutrophils	FCGR3B	0.279	3.2E-11	0.239	3.6E-05	0.274	9.0E-11	0.045	4.5E-01	0.037	5.5E-01	0.048	4.2E-01
	CEACAM3	0.307	2.6E-13	0.283	8.4E-07	0.308	2.5E-13	-0.015	8.0E-01	-0.052	4.1E-01	-0.024	6.8E-01
	SIGLEC5	0.210	7.7E-07	0.204	4.5E-04	0.215	4.6E-07	0.171	3.5E-03	0.146	1.9E-02	0.172	3.6E-03
	FPR1	0.195	4.7E-06	0.187	1.3E-03	0.199	3.1E-06	0.081	1.7E-01	0.037	5.6E-01	0.071	2.3E-01
	CSF3R	0.234	3.2E-08	0.184	1.5E-03	0.248	5.4E-09	0.085	1.5E-01	0.051	4.2E-01	0.091	1.2E-01
	S100A12	0.247	5.4E-09	0.244	2.4E-05	0.249	4.7E-09	-0.087	1.4E-01	-0.113	7.0E-02	-0.079	1.8E-01
Macrophages	CD68	0.189	9.1E-06	0.173	3.0E-03	0.196	4.3E-06	0.390	7.8E-12	0.372	7.1E-10	0.388	1.3E-11
	CD84	0.210	7.9E-07	0.194	8.3E-04	0.216	3.9E-07	0.259	8.2E-06	0.269	1.2E-05	0.266	5.6E-06
	CD163	0.094	2.8E-02	0.092	1.2E-01	0.102	1.7E-02	0.180	2.1E-03	0.173	5.2E-03	0.173	3.5E-03
	MS4A4A	0.094	2.8E-02	0.079	1.8E-01	0.098	2.3E-02	0.162	5.8E-03	0.157	1.1E-02	0.157	7.9E-03
Dendritic cells	CD209	0.077	7.4E-02	0.113	5.3E-02	0.081	5.9E-02	-0.045	4.5E-01	-0.033	6.0E-01	-0.044	4.6E-01
NK cells	KIR3DL3	0.145	7.0E-04	0.121	3.8E-02	0.140	1.1E-03	-0.048	4.1E-01	-0.052	4.0E-01	-0.043	4.7E-01
	NCR1	0.186	1.2E-05	0.137	1.9E-02	0.181	2.3E-05	0.039	5.1E-01	0.032	6.0E-01	0.035	5.6E-01
Th1 cells	TBX21	0.132	2.1E-03	0.123	3.6E-02	0.135	1.7E-03	0.069	2.4E-01	0.056	3.7E-01	0.060	3.2E-01
Treg	FOXP3	0.160	1.7E-04	0.146	1.3E-02	0.155	3.0E-04	-0.095	1.1E-01	-0.110	7.8E-02	-0.085	1.5E-01
	CCR8	0.165	1.1E-04	0.149	1.1E-02	0.158	2.2E-04	-0.014	8.2E-01	-0.046	4.7E-01	-0.007	9.0E-01
Monocyte	C3AR1	0.138	1.2E-03	0.116	4.6E-02	0.143	8.8E-04	0.231	7.6E-05	0.231	1.8E-04	0.231	8.3E-05
	CD86	0.164	1.1E-04	0.161	5.7E-03	0.171	6.6E-05	0.166	4.7E-03	0.153	1.4E-02	0.166	5.0E-03
	CSF1R	0.147	5.6E-04	0.150	1.0E-02	0.150	4.6E-04	0.134	2.3E-02	0.110	7.7E-02	0.132	2.6E-02

UCEC, Uterine Corpus Endometrial Carcinoma; KIRP, Kidney Renal Papillary Cell Carcinoma; NK cells, Natural killer cells; Th 1 cells, type I helper T cells; Treg, regulatory T cells; COR, r value of Spearman’s correlation; Purity, correlation adjusted by purity; Age correlation adjusted by age.

### Prognostic analysis of ACE2 expressions in different tumors based on immune cells

We have confirmed that the expressions of ACE2 were correlated with the immune infiltration in uterine corpus endometrial carcinoma and kidney renal papillary cell carcinoma, and the expressions of ACE2 were also related to the favorable prognoses of these tumors. So we speculated that the expressions of ACE2 in these tumors affected the prognosis partly because of immune infiltration.

**Figure 4:**
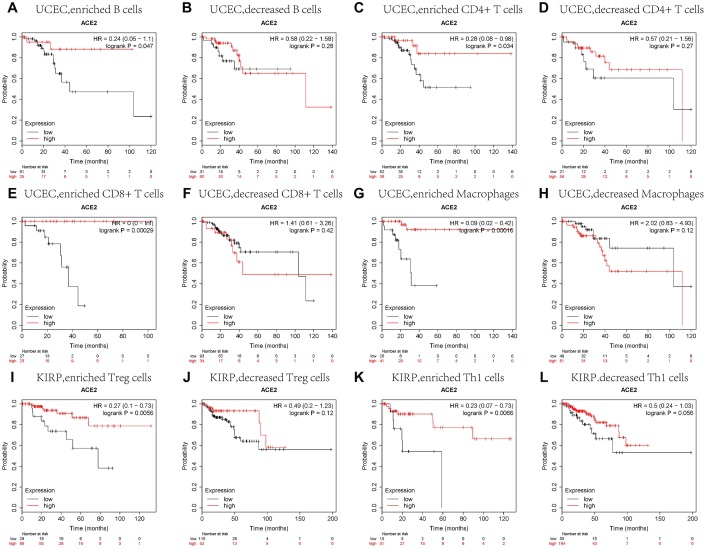
**Comparison of Kaplan-Meier survival curves of the high and low expression of ACE2 in UCEC and KIRP based on immune cells subgroups.** Relationships between ACE2 of different immune cells subgroup and prognoses in (**A**–**H**) Uterine Corpus Endometrial Carcinoma (UCEC), and (**I**–**L**) Kidney Renal Papillary Cell Carcinoma (KIRP).

We did a prognosis analysis based on the expression levels of ACE2 of different tumors in related immune cells subgroup via the Kaplan Meier plotter. The results showed that the high expression of ACE2 of uterine corpus endometrial carcinoma in enriched B cells (HR = 0.24), enriched CD4+ memory T cells (HR = 0.28), enriched CD8+ T cells (HR = 0) and enriched macrophages (HR = 0.09) cohort had better prognosis respectively (Figure 4A, 4C, 4E, 4G). Similarly, the high expression of ACE2 of Kidney Renal Papillary Cell Carcinoma had better prognosis in enriched regulatory T cells (HR = 0.27) and enriched type 1 T helper cells (HR = 0.23) cohort respectively (Figure 4I, 4K). But there was no significant correlation between the high ACE2 and the prognosis of Kidney Renal Papillary Cell Carcinoma in the enriched macrophages cohort (OS HR0.63, 95%CI=0.3 to 1.3, logrank P=0.21), and the high expressions of ACE2 of UCEC and KIRP had no significant correlation in decreased immune cells subgroup (Figure 4B, 4D, 4F, 4H, 4J, 4L). The above analysis suggested that high ACE2 expressions in UCEC and KIRP may affect prognoses in part due to immune infiltration.

### Promoter methylation levels of ACE2 decreased in UCEC and KIRP

**Figure 5:**
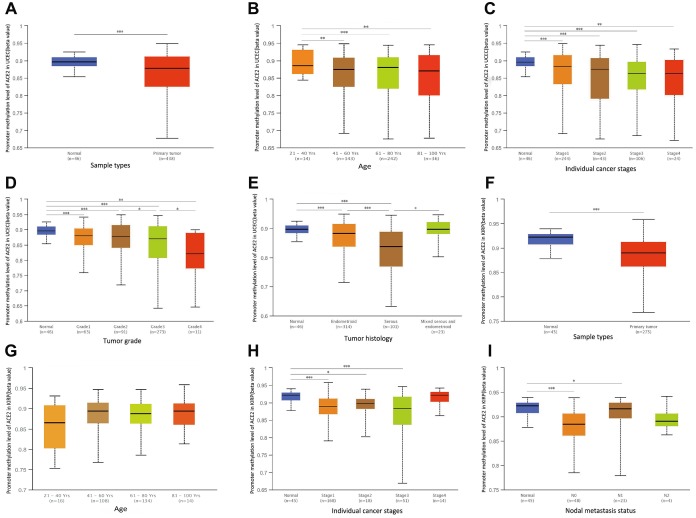
**The promoter methylation levels of ACE2 in UCEC and KIRP.** Promoter methylation levels of ACE2 were low in (**A**–**E**) Uterine Corpus Endometrial Carcinoma (UCEC) and (**F**–**I**) Kidney Renal Papillary Cell Carcinoma (KIRP) (^*^P<0.05, ^**^P<0.01, ^***^P<0.001).

The significant increases of ACE2 expressions in UCEC and KIRP were observed. Therefore, we further studied the reason for the elevated ACE2. DNA methylation is an important event in the epigenetic modification of the genome and is closely related to the process of the disease [33]. In particular, hypomethylation can lead to genome instability [34, 35], and may activate related genes. So we used UALCAN database to verify the methylation levels of ACE2 promoter in UCEC and KIRP. Interestingly, the methylation levels of ACE2 promoter in UCEC and KIRP were significantly lower than that in normal tissue (Figure 5A, 5F). Also, we stratified UCEC and KIRP according to patients' age, individual cancer stages, tumor grade, tumor histology and nodal metastasis status.

The results showed that ACE2 promoter methylation levels of the older people, higher grade tumors and serous tumors groups were lower than control in UCEC (Figure 5B, 5D, 5E). Moreover, the ACE2 promoter methylation levels of tumors with lymph node metastasis group in KIRP were lower than that in normal tissue (Figure 5I). And the ACE2 promoter methylation levels of different individual cancer stage groups decreased significantly compared with normal tissues groups in UCEC and KIRP (Figure 5C, 5H). However, the ACE2 promoter methylation levels of ACE2 did not change significantly in different age subgroups of KIRP and individual cancer stages subgroups of UCEC and KIRP (Figure 5C, 5G, 5H). It is suggested that ACE2 promoter hypomethylation in UCEC and KIRP may activate itself and increase its level respectively.

### SARS-CoV-2 infection may reduce the expression of ACE2

**Figure 6:**
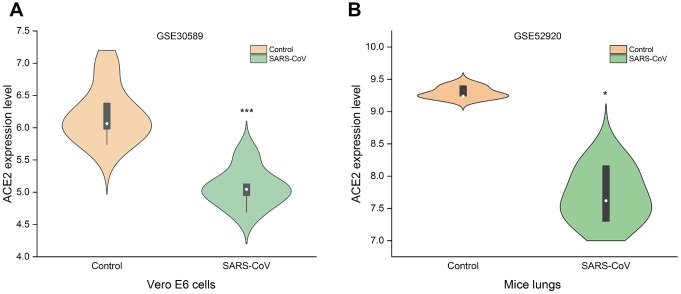
**Changes of ACE2 after SARS-CoV infection.** SARS-CoV reduced the expression levels of ACE2 in (**A**) Vero E6 cells and (**B**) mice lungs (^***^P<0.001, ^*^P<0.05 vs. Control).

ACE2 can be used as a receptor for SARS-CoV-2 to enter the cell [24]. It is necessary to study the changes of ACE2 in tumors after SARS-CoV-2 infection. Because SARS-CoV-2 and SARS-CoV have high homology [24], the change of ACE2 expression after cells or animals infected with SARS-CoV can be used as a reference for SARS-CoV-2. GSE30589 and GSE52920 databases were used to analyze the changes of ACE2 expression after SARS-CoV infected Vero E6 cells and mice lung. The results showed that the expressions of ACE2 in Vero E6 cells and mouse lung decreased significantly compared with control group (Figure 6). This finding suggested that ACE2 expression may decrease after SARS-CoV-2 infection.

## DISCUSSION

In this study, the changes of ACE2 mRNA in UCEC and KIRP were analyzed in Oncomine and TIMER databases. And we analyzed the correlations between ACE2 expression levels and immune infiltration and the prognoses of these tumors. Moreover, we predicted the susceptibility of different tumor tissues to SARS-CoV-2 and the potential prognoses of patients after SARS-CoV-2 infection in UCEC and KIRP.

We analyzed the TCGA database using TIMER database and found that ACE2 was elevated in both UCEC and KIRP (Figure 1B), which suggested that tumor tissues were more likely to be infected with SARS-CoV-2 in UCEC and KIRP. The Kaplan Meier plotter was used to investigate the effect of ACE2 on tumor prognosis, the results showed that high ACE2 had a favorable prognosis in UCEC and KIRP (Figure 2A, 2B). In addition, TIMER database was also used to analyze the correlation between ACE2 and immune infiltration in UCEC and KIRP. The results showed that ACE2 and B cell, CD4 + T cell, neutrophil and dendritic cell infiltration levels were positively correlated in UCEC (Figure 3A). There was also a positive correlation between macrophage infiltration level and ACE2 in KIRP (Figure 3B). The immune cell type markers in UCEC and KIRP were further studied, after correction of tumor purity, ACE2 in UCEC was significantly positively correlated with FCRL2 and MS4A1 in B cells, it was also positively correlated with FCGR3B, CEACAM3, SIGLEC5, CSF3R and S100A12 in neutrophils and CD84 in macrophages, while ACE2 in KIRP was positively correlated with CD68 and CD84 in macrophages (Table 1). These strongly confirmed the positive correlation between ACE2 and immune infiltration in UCEC and KIRP. Prognostic analysis of ACE2 expression levels in different tumor based on immune cells was performed, high ACE2 expression level in UCEC had a favorable prognosis in the enriched B cells, CD4 + memory T cells, CD8 + T cells and macrophages subgroups (Figure 4A–4H), and high ACE2 expression in KIRP had a favorable prognosis in the enriched regulatory T cells and type 1 T helper cells subgroups (Figure 4I, 4K). The analysis suggests that the high expressions of ACE2 in UCEC and KIRP may affect the prognoses of cancer patients in part due to immune infiltration.

In order to explore the causes of elevated ACE2 in UCEC and KIRP, we investigated the level of methylation in UCEC and KIRP. Surprisingly, the promoter methylation levels of ACE2 in UCEC and KIRP were significantly reduced (Figure 5A, 5F). ACE2 may be activated and up-regulated due to its hypomethylation, which to some extent explained the elevated ACE2 in UCEC and KIRP. SARS-CoV-2 can use ACE2 as a receptor to enter cells, and it has high homology with SARS-CoV [24]. Therefore, we used the GSE30589 and GSE52920 databases to study the changes of ACE2 of Vero E6 cells and mouse that were infected with SARS-CoV. The results showed that ACE2 expression levels in both of them were reduced after SARS-CoV infection (Figure 6). This finding suggested that tumor tissues may also have decreased ACE2 levels after SARS-CoV-2 infection in UCEC and KIRP.

In the above analysis, ACE2 was confirmed to be elevated in UCEC and KIRP, Thus, after patients with UCEC and KIRP are infected with SARS-CoV-2, their tumor tissues are more susceptible to virus interference in addition to the respiratory system. Afterwards, tumor tissues infected with SARS-CoV-2 in turn underwent a decrease in ACE2, and reduced ACE2 brought about tumor microenvironment disorders because of reduced immune infiltration, which may worsen the prognoses of UCEC and KIRP patients after SARS-CoV-2 infection.

As an important member of the renin-angiotensin system (RAS), ACE2 has shown different roles in some pathological processes. One study showed that overexpression of ACE2 can protect endothelial cells by inhibiting the inflammatory response, which is beneficial to early prevention of atherosclerosis [36]. Walters et al. [37] found a direct relationship between atrial structural remodeling and plasma ACE2 activity in patients with atrial fibrillation. While ACE2 not only affects the progress of cardiovascular diseases, but also plays a new role in tumor pathology. Yu C et al. [20] found that down-regulating the ACE2/Ang-(1-7)/Mas axis caused breast cancer metastasis by activating store-operated calcium entry (SOCE) and PAK1/NF-κB/Snail1 pathways. ACE2 also down-regulates VEGFa expression in breast cancer cells and inactivates phosphorylation of VEGFR2, MEK1/2 and ERK1/2 in human umbilical endothelial cells, furthermore, ACE2 can prevent breast cancer cell metastasis in zebrafish models [38]. A study carried out by Yu X et al. [39] showed that ACE2 can block the inflammatory response of pancreatic acinar cells by blocking the p38 MAPK/NF-κB signaling pathway. Li J et al. [40] examined the role of ACE2 and FZD1 in squamous cell/adenosquamous carcinoma (SC / ASC) of the gallbladder, the results showed that negative ACE2 expression in SC/ASC was associated with high TNM stage and lymph node metastasis, and survival analysis showed ACE2 and SC/ASC overall survival were positively correlated. ACE2 was also decreased in non-small cell lung cancer (NSCLC), but overexpression of ACE2 in vitro exerted protective effects by inhibiting cell growth and VEGFa production [41]. In addition, ACE2 overexpression in non-small cell lung cancer can inhibit tumor angiogenesis induced by acquired platinum resistance [42]. In general, ACE2 mainly affected tumor metastasis by intervening signaling pathways, but the mechanism by which ACE2 affected the prognosis of UCEC and KIRP is unclear. Here, we found that ACE2 may affect the prognosis of UCEC and KIRP through a new mechanism, that was, immune infiltration, which can provide a direction for future in-depth research. But this research also has some limitations, due to the limitation of the database, we did not continue to analyze the deep relationship between ACE2 and immune infiltration. Also, experiments are urgently needed to verify the analysis results in our research.

## CONCLUSIONS

To sum up, ACE2 expression increased significantly in UCEC and KIRP. Elevated ACE2 was positively correlated with immune infiltration and prognoses of UCEC and KIRP. Moreover, tumor tissues were more susceptible to SARS-CoV-2 infection in COVID-19 patients with UCEC and KIRP. In the end, tumor tissues infected with SARS-CoV-2 may undergo a decrease in ACE2, and reduced ACE2 can bring about reduced immune infiltration in the tumor microenvironment, which may worsen the prognosis of COVID-19 patients with UCEC and KIRP.

## MATERIALS AND METHODS

### Oncomine database analysis

ACE2 expression in different tumors was identified in the Oncomine database (https://www.oncomine.org/resource/main.html) [27]. The threshold was a P-value of 0.01, a 1.5-fold change, and a top 10% of gene ranking. The data must come from mRNA.

### Survival analysis in PrognoScan, GEPIA and Kaplan-Meier plotter databases

To analyze the prognosis of ACE2 expression in various tumors, PrognoScan (http://dna00.bio.kyutech.ac.jp/PrognoScan/index.html) [28], GEPIA (http://gepia.cancer-pku.cn/) [29] and Kaplan-Meier plotter (http://kmplot.com/) [30] databases were used separately. This threshold was cox p-value<0.05 in PrognoScan database, logrank p value <0.05 in GEPIA and Kaplan-Meier plotter database.

### TIMER database analysis

TIMER is a comprehensive database that can analyze the levels of immune invasion in different tumors and the differences in gene expression of different tumors (https://cistrome.shinyapps.io/timer/) [31]. We confirmed the expression of ACE2 in various tumors using the TIMER database. Then the correlation of ACE2 with immune infiltration (B cells, CD4 + T cells, CD8 + T cells, Neutrophils, Macrophages, and Dendritic cells) in the tumor was estimated using the TIMER algorithm. Finally, the correlation of ACE2 with the type markers of B-cells, CD8 + T cells, neutrophils, macrophages, dendritic cells NK cells, Th1 cells, Treg cells and monocytes in UCEC and KIRP were verified. In addition, we used tumor purity and patient's age for p-value correction.

### UALCAN database analysis

UALCAN is a comprehensive interactive web resource for analyzing cancer OMICS data (http://ualcan.path.uab.edu/index.html) [32]. It is built on PERL-CGI and can be used to assess the methylation levels of different genes. So the ACE2 promoter methylation profile was tested in the UALCAN database. Moreover, we performed a stratified analysis based on patients' age, individual cancer stages, tumor grade, tumor histology and nodal metastasis status.

### Microarray data collection

GEO (https://www.ncbi.nlm.nih.gov/geo/) is a public repository that can archive microarrays and other forms of high-throughput functional genomics data, and the expression profiles of GSE30589 and GSE52920 were obtained in the GEO database. The GSE30589 database which contained 12 SARS-CoV infected samples and 9 control samples was based on the GPL570 platform ([HG-U133_Plus_2] Affymetrix Human Genome U133 Plus 2.0 Array). While the GSE52920 database which included 3 lung tissue samples of mice infected with SARS-CoV and 3 normal mice lung tissue samples was based on the GPL13912 platform (Agilent-028005 SurePrint G3 Mouse GE 8x60K Microarray).

### Statistical analysis

The statistical results of the survival analysis were obtained from a log-rank test, and the correlations of ACE2 with immune infiltration and type markers of immune cells were evaluated using Spearman’s correlation. Student's t test was used to compare two independent samples. p-values less than 0.05 were considered statistically significant.

## Supplementary Material

Supplementary Figures

Supplementary Tables
