# Supplementary material for: ACE2 correlated with immune infiltration serves as a prognostic biomarker in endometrial carcinoma and renal papillary cell carcinoma: implication for COVID-19
Source: Aging (Albany NY). 2020 Apr 27;12(8):6518–35. doi: 10.18632/aging.103100 (PMC7202533; doi:10.18632/aging.103100)
Supplement: Supplementary Figures [file aging-12-103100-s002..pdf]

SUPPLEMENTARY FIGURES

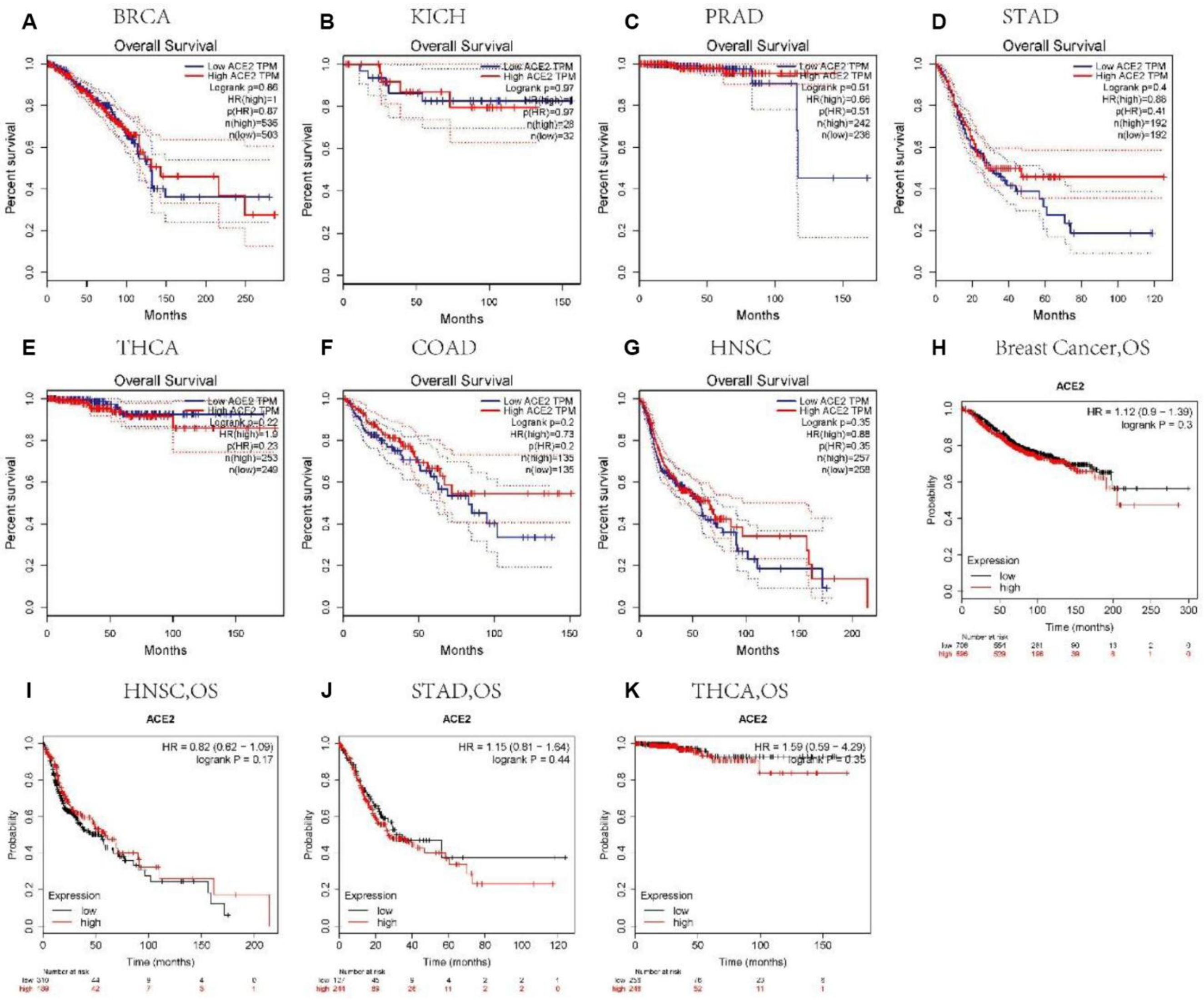

Supplementary Figure 1. Correlations between ACE2 expression and immune infiltration in different cancers. Comparison of overall survival of ACE2 over-expression and under-expression in (**A**–**G**) Breast Invasive Carcinoma (BRCA), Kidney Chromophobe (KICH), Prostate Adenocarcinoma (PRAD), Stomach Adenocarcinoma (STAD), Thyroid Carcinoma (THCA), Colon Adenocarcinoma (COAD) and Head and Neck Cancer (HNSC) in GEPIA database, (**H**–**K**) Breast Cancer, Head and Neck Cancer (HNSC), Stomach Adenocarcinoma (STAD) and Thyroid Carcinoma (THCA) in Kaplan Meier plotter database.

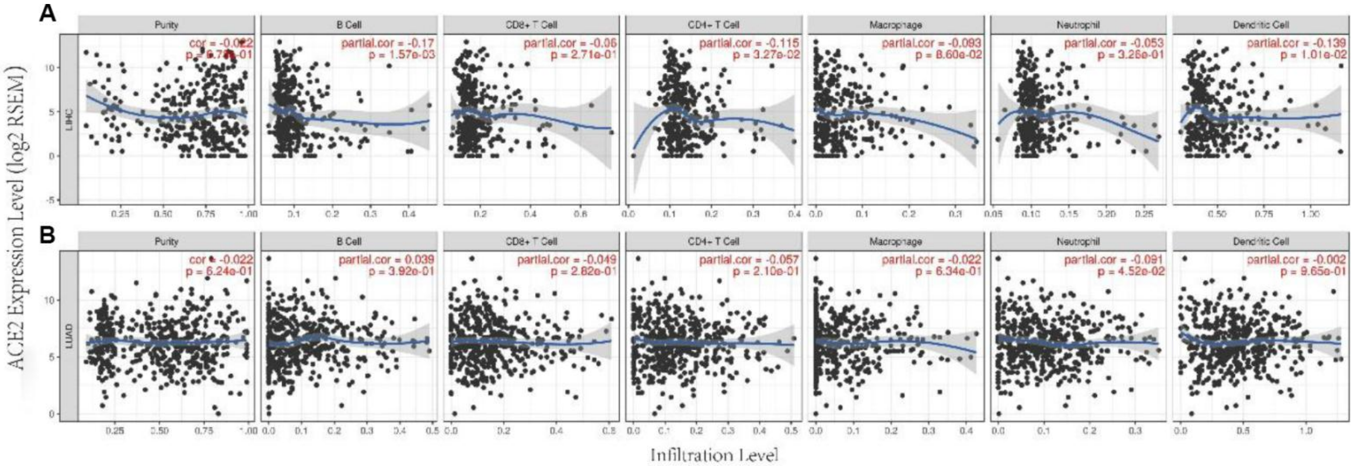

Supplementary Figure 2. Correlation between ACE2 expression and immune infiltration in LIHC and LUAD in TIMER database. ACE2 was very weakly negatively correlated with (**A**) B cell, CD4 + T cell, dendritic cell of Liver hepatocellular carcinoma (LIHC), and (**B**) neurophil of lung adenocarcinoma (LUAD).
