# Supplementary material for: ACE2 correlated with immune infiltration serves as a prognostic biomarker in endometrial carcinoma and renal papillary cell carcinoma: implication for COVID-19
Source: Aging (Albany NY). 2020 Apr 27;12(8):6518–35. doi: 10.18632/aging.103100 (PMC7202533; doi:10.18632/aging.103100)
Supplement: Supplementary Tables [file aging-12-103100-s001..pdf]

SUPPLEMENTARY TABLES

Supplementary Table 1. The expression levels of ACE2 in Oncome database compared with normal tissues.

Cancer	Cancer subtype	P-value	Fold change	Rank (%)	Sample	Reference (PMID)
Breast cancer	Invasive Breast Carcinoma	2.11E-16	2.279	4%	725	18438415
	Invasive Ductal Breast Carcinoma	1.11E-4	-1.751	2%	324	19187537
	Invasive Lobular Breast Carcinoma	1.93E-11	-3.134	5%	931	TCGA
	Invasive Ductal Breast Carcinoma	9.75E-26	-2.684	6%	1124	TCGA
	Intraductal Cribriform Breast Adenocarcinoma	0.008	-5.450	10%	2016	TCGA
colorectal cancer	Colon Mucinous Adenocarcinoma	9.11E-5	-2.690	8%	1413	17615082
	Colon Adenoma	8.55E-5	-3.739	9%	1650	20957034
Esophageal Cancer	Barrett's Esophagus	1.21E-4	3.893	10%	1820	21152079
	Esophageal Adenocarcinoma	0.002	-5.143	3%	445	16952561
	Barrett's Esophagus	0.006	-2.947	4%	560	16952561
Head and Neck cancer	Tongue Squamous Cell Carcinoma	0.002	1.537	10%	1763	18254958
kidney cancer	Renal Wilms Tumor	4.08E-4	-64.062	1%	156	20440404
	Chromophobe Renal Cell Carcinoma	0.003	-21.769	4%	779	20440404
	Renal Oncocytoma	0.003	-13.376	5%	958	20440404
	Chromophobe Renal Cell Carcinoma	1.50E-10	-5.131	2%	212	16115910
	Renal Pelvis Urothelial Carcinoma	1.12E-6	-3.856	7%	841	16115910
	Renal Oncocytoma	1.17E-7	-4.528	9%	1026	16115910
	Renal Wilms Tumor	0.005	-1.959	7%	843	16299227
	Clear Cell Sarcoma of the Kidney	0.010	-2.843	10%	1255	16299227
Liver cancer	Cirrhosis	5.12E-4	2.842	5%	874	17393520
	Liver Cell Dysplasia	0.005	1.956	5%	969	17393520
	Cirrhosis	6.51E-9	1.755	9%	1054	19098997
Lung cancer	Lung Adenocarcinoma	1.36E-11	2.039	5%	866	23028479
Lymphoma	Germinal Center B-Cell-Like Diffuse Large B-Cell Lymphoma	2.94E-11	-1.872	1%	118	19412164, 19965633, 21156281, 21390126, 22137796, 28314854, 28288979
	Activated B-Cell-Like Diffuse Large B-Cell Lymphoma	1.06E-10	-1.841	3%	556	
	Follicular Lymphoma	1.81E-9	-1.649	9%	1568	
	Diffuse Large B-Cell Lymphoma	2.15E-10	-1.809	10%	1800	
Other cancer	Testicular Intratubular Germ Cell Neoplasi	0.009	4.922	2%	170	15994931
	Yolk Sac Tumor	1.61E-12	-17.251	1%	25	16424014, 25336442, 26624623
	Seminoma	3.01E-12	-14.554	1%	46	
	Mixed Germ Cell Tumor	1.48E-19	-12.891	1%	50	
	Embryonal Carcinoma	2.54E-10	-13.188	2%	282	
	Testicular Embryonal Carcinoma	3.39E-4	-23.719	4%	477	15994931
	Testicular Yolk Sac Tumor	6.09E-4	-20.922	4%	554	
	Testicular Seminoma	0.001	-30.784	6%	809	
	Uterine Corpus Leiomyoma	3.01E-4	-1.641	5%	799	19622772
	Malignant Fibrous Histiocytoma	0.003	-3.649	9%	1058	15994966, 16603191
pancreatic cancer	Pancreatic Ductal Adenocarcinoma	0.006	-2.373	2%	333	16053509

sarcoma	Malignant Fibrous Histiocytoma	0.003	-3.649	9%	1058	15994966, 16603191
	Clear Cell Sarcoma of the Kidney	0.010	2.843	10%	1255	16299227
	Gastrointestinal Stromal Tumor	8.51E-5	-3.067	7%	1335	21447720, 29725014, 23112551

Supplementary Table 2. Relationships between ACE2 expressions and prognoses in different cancers in PrognoScan database.

DATASET	CANCER TYPE	SUBTYPE	ENDPOINT	N	COX P-VALUE	HR [95% CI-low CI-upp]
GSE9893	Breast cancer	-	Overall Survival	155	0.207469	0.83 [0.61 - 1.11]
GSE1456-GPL96	Breast cancer	-	Overall Survival	159	0.749893	1.05 [0.78 - 1.40]
GSE1456-GPL96	Breast cancer	-	Overall Survival	159	0.24964	1.28 [0.84 - 1.93]
E-TABM-158	Breast cancer	-	Overall Survival	117	0.263366	1.39 [0.78 - 2.47]
E-TABM-158	Breast cancer	-	Overall Survival	117	0.531078	1.19 [0.69 - 2.07]
GSE7390	Breast cancer	-	Overall Survival	198	0.002524	1.23 [1.08 - 1.41]
GSE7390	Breast cancer	-	Overall Survival	198	0.006852	1.18 [1.05 - 1.33]
GSE12945	Colorectal cancer	-	Overall Survival	62	0.081404	0.68 [0.44 - 1.05]
GSE12945	Colorectal cancer	-	Overall Survival	62	0.112689	0.68 [0.43 - 1.09]
GSE17536	Colorectal cancer	-	Overall Survival	177	0.841356	0.98 [0.82 - 1.18]
GSE17536	Colorectal cancer	-	Overall Survival	177	0.929774	0.99 [0.85 - 1.16]
GSE17537	Colorectal cancer	-	Overall Survival	55	0.592087	1.11 [0.75 - 1.64]
GSE17537	Colorectal cancer	-	Overall Survival	55	0.494492	1.11 [0.82 - 1.49]
GSE11595	Esophagus cancer	Adenocarcinoma	Overall Survival	34	0.974499	0.98 [0.38 - 2.53]
GSE2837	Head and neck cancer	Squamous cell carcinoma	Relapse Free Survival	28	0.308794	1.75 [0.60 - 5.12]
GSE11117	Lung cancer	NSCLC	Overall Survival	41	0.118476	1.37 [0.92 - 2.03]
GSE3141	Lung cancer	NSCLC	Overall Survival	111	0.362302	0.87 [0.63 - 1.18]
GSE3141	Lung cancer	NSCLC	Overall Survival	111	0.542736	0.91 [0.68 - 1.22]
GSE14814	Lung cancer	NSCLC	Overall Survival	90	0.973271	1.02 [0.35 - 2.93]
GSE14814	Lung cancer	NSCLC	Overall Survival	90	0.442885	1.52 [0.52 - 4.42]
GSE4573	Lung cancer	Squamous cell carcinoma	Overall Survival	129	0.809129	0.94 [0.56 - 1.57]
GSE17710	Lung cancer	Squamous cell carcinoma	Overall Survival	56	0.149829	0.76 [0.52 - 1.10]

GSE17710	Lung cancer	Squamous cell carcinoma	Overall Survival	56	0.104661	0.73 [0.50 - 1.07]
E-DKFZ-1	Renal cell carcinoma	-	Overall Survival	59	0.021041	0.17 [0.04 - 0.77]
